# Supplementary material for: Agave Fructans in Oaxaca’s Emblematic Specimens: Agave angustifolia Haw. and Agave potatorum Zucc
Source: Plants (Basel). 2022 Jul 13;11(14):1834. doi: 10.3390/plants11141834 (PMC9319672; doi:10.3390/plants11141834)
Supplement: Supplementary file 1 [file plants-11-01834-s001.zip › plants-1720693-supplementary.pdf]

Table S1. Comparative analysis (Tukey α=0.05) of morphological parameters of *A. angustifolia* Haw. plants of different ages.

Age (years)	**PL**	**RD**	**NL**	**LL**	**LW**	**NS**	**ASL**	**MLD**	**MTD**	**MED**
1	39.00±1.73^{a}	57.66±1.45^{a}	13.66±0.88^{a}	36.66±2.72^{a}	4.83±0.166^{a}	41.33±0.66^{a}	2.33±0.08^{a}	33.33±0.88^{a}	37.33±0.33^{a}	73.66±0.88^{a}
2	84.33±0.88^{b}	122.66±1.46^{b}	24.33±0.88^{b}	60.33±1.45^{b}	6.16±0.166^{b}	47.33±0.66^{b}	2.40±0.05^{a}	42.33±0.89^{b}	45.00±0.57^{b}	92.33±1.45^{b}
3	127.00±1.52^{c}	182.33±1.20^{c}	33.00±0.57^{c}	101.00±2.66^{c}	7.66±0.33^{b}	68.66±0.70^{c}	2.40±0.05^{a}	85.66±1.85^{c}	93.00±1.52^{c}	186.66±1.20^{c}
4	134.00±2.08^{c}	203.00±1.52^{d}	42.66±1.45^{d}	115.33±1.45^{c}	11.00±0.57^{c}	82.00±1.15^{d}	2.76±0.38^{a}	94.66±0.33^{d}	107.33±1.20^{d}	207.00±1.73^{d}
5	153.00±1.73^{d}	229.33±2.96^{e}	57.00±0.57^{e}	124.33±0.88^{d}	13.00±0.58^{d}	83.33±0.67^{d}	2.60±0.05^{a}	104.00±0.57^{e}	125.00±2.51^{e}	241.33±0.88^{e}
6	220.66±1.85^{e}	309.33±1.76^{f}	73.00±2.64^{f}	160.00±2.88^{e}	15.66±0.88^{e}	96.00±0.57^{e}	2.46±0.03^{a}	133.66±0.33^{f}	142.00±1.15^{f}	282.66±1.45^{f}

PL= plant length, RD= rosette diameter, NL=number of leaves, LL= leaf length, LW= leaf width, NS=number of spines, ASL=Apical spine length, MLD= mezontle longitudinal diameter, MTD=mezontle transversal diameter, MED= mezontle equatorial diameter. Mean value ± standard error. Measurements are in cm, except NL and NS, which were counted. Different letters indicate significant differences with $P<0.05$.

Table S2. Comparative analysis (Tukey α=0.05) of morphological parameters of *A. potatorum* Zucc. plants of different ages.

Age (years)	**PL**	**RD**	**NL**	**LL**	**LW**	**NS**	**ASL**	**MDL**	**MTD**	**MED**
1	12.40±0.40^{a}	22.60±1.12^{a}	10.80±0.48^{a}	12.00±2.38^{a}	6.30±0.38^{a}	11.20±0.63^{a}	1.90±0.11^{a}	4.60±0.24^{a}	4.00±0.54^{a}	14.26±3.66^{a}
2	26.00±1.05^{b}	34.00±0.57^{b}	15.00±0.57^{b}	20.00±1.15^{b}	11.66±0.88^{b}	22.66±1.76^{b}	4.33±0.33^{a}	8.16±0.44^{b}	8.83±0.44^{b}	33.33±0.33^{b}
3	27.00±0.57^{c}	43.66±0.33^{c}	18.66±0.33^{c}	27.33±1.45^{c}	17.66±0.33^{c}	25.33±2.66^{c}	3.33±0.35^{a}	8.66±0.33^{b}	8.90±0.45^{b}	34.00±0.57^{b}
4	30.00±1.15^{d}	43.77±0.66^{c}	25.66±0.35^{d}	31.00±1.02^{d}	26.33±0.88^{d}	31.66±0.88^{d}	2.83±0.16^{a}	12.00±0.57^{c}	11.00±0.57^{c}	42.50±3.27^{c}
5	34.00±0.57^{e}	57.33±1.45^{d}	33.33±0.66^{f}	34.00±0.57^{d}	31.00±0.57^{f}	35.33±0.90^{e}	3.00±0.01^{a}	13.66±0.33^{c}	14.00±0.57^{d}	52.33±0.33^{d}
6	47.33±0.66^{f}	84.66±1.45^{f}	35.00±0.57^{f}	43.16±1.69^{e}	44.33±0.66^{g}	42.33±1.45^{f}	3.66±0.33^{a}	15.00±0.57^{d}	18.33±0.66^{f}	63.66±0.88^{f}

PL= plant length, RD= rosette diameter, NL=number of leaves, LL= leaf length, LW= leaf width, NS=number of spines, ASL=Apical spine length, MLD= mezontle longitudinal diameter, MTD=mezontle transversal diameter, MED= mezontle equatorial diameter. Mean value ± standard error. Measurements are in cm, except NL and NS, which were counted. Different letters indicate significant differences with P<0.05.

Table S3. Variables and three principal components (PC) of morphological parameters of *A. angustifolia* Haw. and *A. potatorum* Zucc. plants of different ages.

Variable	CP1	CP2	CP3
PL	**0.3590**	0.0044	0.0342
RD	**0.3613**	0.0491	-0.0035
NL	**0.2978**	**0.3677**	-0.3121
LL	**0.3617**	-0.0156	0.1203
LW	-0.0629	**0.6656**	-0.4818
NS	**0.3442**	0.1786	-0.0535
ASL	-0.1050	**0.5996**	0.7875
MLD	**0.3574**	-0.1175	0.1058
MTD	**0.3566**	-0.1164	0.1174
MED	**0.3618**	0.0103	0.0811

PL= plant length, RD= rosette diameter, NL=number of leaves, LL= leaf length, LW= leaf width, NS=number of spines, ASL=Apical spine length, MLD= mezontle longitudinal diameter, MTD=mezontle transversal diameter, MED= mezontle equatorial diameter.
